# Age and moral disgust: An experimental priming effects vignette study

**DOI:** 10.1371/journal.pone.0328392

**Published:** 2025-08-27

**Authors:** Guido Corradi, Pilar Aguilar, Fernando Aguiar, Antonio Olivera-La Rosa

**Affiliations:** 1 BEATLES Research Group, University of Balearic Islands, Illes Balears, Spain; 2 Facultad de Medicina, Universidad Complutense de Madrid, Madrid, Spain; 3 Department of Social Psychology, Facultad de Psicología, Universidad de Sevilla, Sevilla, Spain; 4 Institute of Philosophy, CSIC, Madrid, Spain; 5 Department of Psychology, Basic and Applied Neuroscience Group, Universidad Católica Luis Amigó, Medellín, Colombia; 6 Human Evolution and Cognition Group, University of the Balearic Islands, Palma de Mallorca, Spain; Pacific Lutheran University, UNITED STATES OF AMERICA

## Abstract

Disgust influences how people perceive and judge moral violations. Individuals who are more sensitive to disgust often judge such violations more harshly. This sensitivity may change depending on the context, like the age of the person committing the act. However, there’s limited and inconsistent evidence on how disgust sensitivity and feelings of disgust affect moral judgments toward older adults. This study aimed to find out if disgust sensitivity and induced feelings of disgust affect moral judgments differently based on the age of the person involved. Specifically, we examined whether people relate their disgust sensitivity differently when judging actions by young versus old individuals and whether showing disgusting images influences the harshness of moral judgments differently depending on the actor’s age. We conducted a preregistered experiment with 235 adult participants. Participants were randomly assigned to one of two groups: “Old” or “Young.” They read 24 stories about moral violations committed by either old or young individuals. Before each story, participants were briefly shown an image that was either disgusting, sad, or neutral. They then rated the acceptability of the behavior on a scale. Participants also filled out questionnaires measuring their disgust sensitivity, attitudes toward age, and emotions toward young and old people. The findings showed that higher disgust sensitivity was linked to harsher moral judgments, regardless of the age of the person committing the violation. This means that how sensitive someone is to disgust affects their moral judgments in the same way for both young and old actors. However, when participants were shown disgusting images before reading about an older person committing a moral violation, they judged the behavior less harshly compared to when the actor was young. This effect was not seen with sad or neutral images. There was also no significant interaction between the type of image shown and the participant’s disgust sensitivity. The study suggests that disgust has a complex role in moral judgments. While personal sensitivity to disgust leads to harsher judgments no matter who is involved, feelings of disgust induced by images can influence judgments differently based on the age of the person committing the act.

## 1 Introduction

The latest advances in the field suggest that disgust is involved in certain aspects of moral psychology [1–3]. Also, people are known to judge the actions of old people differently to those of young people [4,5]. Previous work in a vignette study and correlational research design [6] has found that disgust sensitivity affects how harshly moral violations of old adults are judged. However, evidence on the relationship between disgust sensitivity and moral judgments (i.e., judgments of a moral violation) towards the elderly is still scarce and requires further experimental studies. Building on the reviewed literature, the present research is intended to extend current research in this largely unexplored topic, by examining whether the influence of affective priming of disgusting pictures on moral judgments is sensitive to the target’s age.

The study of the cognitive/affective foundations of stigmatization indicates that disgust is a relevant variable in this process [3,7,8]. Disgust is at its core an emotional response that is elicited by cues of parasites and disease, experienced as a sense of revulsion, offense, notions of pollution, and a behavioral tendency to avoid a diverse pool of stimuli [9,10]. Disgust sensitivity (i.e., the degree to which people experience disgust) varies remarkably across individuals, cultures, and geographical regions [11–13]. There is evidence that disgust sensitivity decreases with age [14] and that women tend to be more sensitive to disgust than men [12,15]. Disgust sensitivity is associated with several areas of psychology, such as personality traits, psychiatric disorders, and social belief systems [16,17].

Although it is widely held that disgust evolved as a pathogen avoidance mechanism [18,19], some authors argued that disgust has come to be co-opted to be a sociomoral emotion, extending its original function to also protect the psyche from moral degradation [12,20]. Several studies suggest that disgust is involved in certain aspects of moral cognition [1–3]. Disgust can exert a causal role in moral judgments (i.e., judgments of a moral violation), can be a consequence of moral violations, or can “moralize” otherwise neutral acts and persons [21,22]. For instance, some studies found that disgust experienced incidentally makes moral judgments harsher [23,24], although this influence may be susceptible to certain moderators [25,26], such as individual sensitivity to bodily cues [27]. While recent studies have questioned the robustness of the link between incidental disgust and moral judgment [28–31], some authors highlight that more work is needed to determine which disgust inductions, if any, consistently produce carry-over effects on moral judgment [32].

Some authors claim that disgust triggered by a subclass of egregious moral violations (e.g., enjoyment of child pornography) can “degrade” the perpetrator, functioning as a guard against such actions being carried out [20]. Giner-Sorolla and colleagues [3] suggest that disgust not only helps people to value what is “good” or what is “rotten” socially, but also informs perceived intentions and characteristics of the social actors, especially judgments of moral character. Indeed, moral evaluations are not context-free, but are often influenced by the focus on the person being judged [33–34] Research by Giner-Sorolla et al. [3] and Uhlmann et al. [34] suggests that incidental disgust might have a stronger influence on evaluations of individuals, especially when morally irrelevant aspects, such as age, are considered. Since the disgusting is perceived as degraded and polluting [40], disgust establishes an implicit order in which the target of the repugnant is regarded as inferior and impure [35–37; 7]. Consistent with this, disgust influences negative attitudes to a variety of outgroups, such as immigrants, foreigners, socially deviant groups [8,40], obese people [41], and gay men [38,39]. Disgust, then, is an emotion with a great capacity to stigmatize, i.e., rejecting not just individuals but entire social groups [40,41].

One social group that is often included in the realm of disgust is older adults [5,42]. Some evidence suggests that older adults do not necessarily provoke disgust by themselves, but as representatives of old age per se [43]. That is, disgust towards old adults may be explained because of biases about aging as a whole. Perception of vulnerability to disease and awareness of ageing may contribute to this negative bias [44]. Thus, old age is rejected as much as it could transmit illnesses and, what is more, remind us of our mortality [45–47]. Although ageism (i.e., negative attitudes towards older adults) is pervasive in many societies, more research is needed on its correlates and predictors [48]. A consistent finding is that males are more ageist than females [49,50]. Ageing anxiety (anxiety about getting older) has been related to ageism in several studies [49,51,52, but see [53]. Some authors claim that ageism in younger people should be explained as part of a coping strategy to deal with unpleasant thoughts of mortality [54].

Despite anecdotal evidence supporting this link, empirical evidence on disgust towards old adults remains limited and offers mixed results. Nicol and his colleagues [48] found that prejudice towards older adults is related to disgust sensitivity, but that this relationship is mediated by perceived vulnerability to disease and ageing anxiety. A recent study [6] found that older adults were judged less morally harshly than younger adults, with higher disgust sensitivity being associated with harsher moral judgments, irrespective of the age of the perpetrator. Interestingly, lower disgust sensitivity was associated with less harsh (less severe) moral judgments towards older people than to younger people, (e.g.,) suggesting that low disgust sensitivity could partially underlie compassionate paternalism towards the elderly. Altogether, actual empirical research on the role of disgust in moral judgments toward the elderly suggest that this relationship may be more complex (e.g., sensitive to moderators) than predicted by theoretical assumptions and anecdotal evidence.

The fact that the influence of incidental affective responses on moral judgments appears to be largely automatic [55,56] encourages affective priming as a relevant tool for the study of moral cognition. This experimental paradigm is built on the assumption that the affective nature of the prime (e.g., a disgusting image) will impact the subject’s evaluation of the target stimulus (e.g., a moral dilemma). Indeed, affective priming often occurs independently of evaluative intention and of awareness of the prime [57]. Although affective priming has been effectively applied to a vast number of studies [58], this technique has scarcely been applied to moral research. There is evidence that erotic pictures presented in a suboptimal manner (but not other types of pleasant pictures) increase utilitarian moral judgments, which might be due to the erotic stimuli being more arousing [59]. Affective priming through disgusting pictures (depicting human mutilation) exclusively reduced the severity of moral judgments (with no effect on non-moral judgments [60]). The effect of disgust priming on moral judgments may be moderated by how sensitive the individual is to disgust: while disgusting pictures favoured utilitarian judgments in participants with higher disgust sensitivity, the same pictures favoured deontological judgments in participants with lower disgust sensitivity [61]. In sum, the few studies conducted on the effects of affective priming on moral judgments show mixed results, suggesting that more research is needed to disambiguate the direction of the effects of affective priming on the harshness of moral judgments. For instance, whether affective priming of disgusting pictures would have a different effect on the harshness of moral judgments than other types of affective pictures (i.e., depicting stimuli associated with different emotional responses) and whether this effect varies depending on the age of the person performing the action requires further research.

Building on the reviewed literature, which suggests that moral evaluations are often influenced by the focus on the person being judged [33–34], and proposing a stronger role for incidental disgust stemming from morally irrelevant aspects of the person or act in question [3], the present research aims to conceptually replicate and extend Aguiar and his colleagues’ [6] finding that disgust sensitivity responds to the target’s age in the making of moral judgments, by examining whether the influence of affective priming of disgusting pictures on moral judgments is sensitive to the target’s age in a pre-registered protocol. Consequently, we propose the following research questions (RQ) summarized in the following Table 1.

**Table 1:** Preregistered research questions.

RQ	Explanation	Design decision	Statistical tests and expectations
1	To investigate the role of disgust sensitivity in judging two age scenarios (characterized by old or young actors) more or less morally harshly.	Manipulation between participants is actors`age (young vs old).	We expect that higher disgust sensitivity is related to harsher (i.e., more severe) moral judgments. We expect the relation between disgust sensitivity and harshness of moral judgments to be stronger in the old aged vignettes relative to young ones. We expect the less-disgust sensitive participants will judge the old themed vignettes less morally harsh than young actor vignettes. Highly disgust-sensitive participants will evidence harsher moral judgments, regardless of the age of the vignette actor
2	Investigate the effect of affective pictures (primes) on the harshness of moral judgments	Within participants manipulation with affective pictures (brief emotionally valenced images with sad, neutral and disgust primes)	We expect that disgust pictures will have a different effect on the harshness of moral judgments than other affective pictures We made no prediction about the direction of the relationship, as explained above, the literature on affective priming and moral judgments could support either a positive or negative difference

## 2 Materials and methods

### 2.1 Procedure

This study used a 2 x 3 mixed design. Participants were randomly assigned to either an “Old” or “Young” condition, representing a between-subjects factor. Within each condition, participants viewed 24 moral vignettes, each preceded by one of three types of affective primes (sad, disgust, or neutral), representing the within-subjects factor.

Participants were recruited through Prolific™. The recruitment period for this study began on November 29, 2023, and concluded on December 3, 2023. Before the study began, the participants completed an online consent form. They completed the study online using JATOS platform with OpenSesame Online software. They were unaware of their assigned condition to minimize bias. In each trial, participants read a moral vignette, followed by a briefly displayed (16ms) affective prime and a white noise mask (100ms). They then rated the acceptability of the behavior described in the vignette on a 7-point scale, with lower scores indicating harsher moral judgments. The vignettes covered four types of moral violations (fairness, care, authority, and sanctity) and differed between conditions only in the age of the main character. The order of vignettes and the pairing with affective primes were randomized.

After completing the main task, participants filled out demographic information and measures on ageism, disgust sensitivity, and emotions towards young and old people. Randomization of participants to conditions and trial order was achieved using JavaScript code integrated into the experimental software. This streamlined design allowed for efficient data collection while maintaining experimental control and enabling the examination of how age and affective primes influence moral judgments.

The project and the experimental protocol were approved by the Ethical Committee of the Research Ethics Committee at the Virgen del Rocío University Hospital (Seville, Spain (approval number: 0770-N18)

### 2.2 Measures and experimental manipulations

#### 2.2.1 Actors’ age.

We manipulated the age of the vignette actor (see [6]). Participants were presented with 24 vignettes, six for each moral foundation proposed by Haidt [55,56]: Care/Harm, Fairness/Cheating, Authority/Subversion and Sanctity/Degradation. These vignettes, describing behavior that violates a specific moral foundation, have already been used in previous studies [6]. In The Old condition, participants were informed that the actor presented in the vignette was an old person whereas, in the Young Condition, the actor of the action was categorized as a young person. We selected the vignettes based on our being able to adapt them to our purposes, that is, if the main character could be substituted by an old or a young character. One example vignette of the old condition is “*You see an old man laughing as he passes a cancer patient with a bald head*”. The list is available in the The Open Science Framework. Repository (https://osf.io/mh8w2/).

#### 2.2.2 Priming.

We manipulated the type of affective priming by means of briefly presented pictures following the procedure described in Olivera- La Rosa et al [58,59]. There are three levels of this categorical variable: Disgust Priming, Sad Priming, and Control (neutral) priming. Each priming condition contains 8 stimulus and each of them is presented for 16ms. The 16 stimuli corresponding to disgust and sadness were taken from Binyamin-Suissa et al. [59]. The eight neutral pictures were taken from the IAPS [61] picture database. The full list is available in the Online Supplementary Repository (https://osf.io/mh8w2/).

#### 2.2.3 Disgust sensitivity.

Participants completed the 27-item Disgust Scale-Revised-Spanish (DS-RS) [62–64] which is divided into three sections: a “core disgust” section comprising twelve items (measuring food-related disgust, animal-related disgust, and disgust related to body products), an “animal reminder” section comprising eight items (measuring disgust towards death and envelope violations), and a “contamination” section comprising five items (measuring concerns about interpersonal transmission). All items were rated on 5-point scales (0–4), where 0 = “*Strongly disagree*” and 4 = “*Strongly agree (very true about me)*”. The scale also included two control-items to identify those participants who were not completing the task properly.

#### 2.2.4 Ageism.

Stereotypes about aging was measured among adults using the CENVE Negative Stereotypes Towards Aging Questionnaire [65,66]. This questionnaire consisted of 15 Likert-type questions, ranging from 1 to 4 (1 = “*Strongly disagree”*, 4 = “*Strongly agree”*).

#### 2.2.5 Emotions.

Participants were asked to report to what extent a young or an old person, depending on the condition, elicit following emotions related to the moral foundations: Gratitude, Guilt, Anger, Compassion, Pride, Rage, Respect, Fear, and Disgust. The scale responses were provided on a 7-point scale ranging from 1 = “Not at all”, to 5 = “Extremely [67]. For example, in the old condition were: “Rate from 1 (not at all) to 5 (extremely) the emotions that best reflect your feelings toward the word OLD”.

#### 2.2.6 Open response.

Participants were asked about which rationale they think underlie the study with an open response option at the end of the study and if they followed any strategy to answer. Also, any additional comment was welcomed. Specifically, we included the following questions: “What do you believe is the purpose of this study?”, “Did you employ any particular strategy when answering the questions?” and “Do you have any other comments?”.

### 2.3 Analysis plan

As we planned, we used generalized linear mixed effects models to analyze the effects of conditioning on moral judgments and the impact of affective pictures. This approach accounts for both between-subject and within-subject effects of the independent variables.

Our main model predicted the harshness of moral judgment based on the interaction between condition (young vs old) and disgust sensitivity score, as well as the interaction between prime type (sad, neutral, disgust) and condition. Participants, items, and primes were set as random effects. We have used the Satterthwaite method to assess coefficient significance and confidence intervals.

Continuous variables were mean-centered. The “Young condition” and “neutral prime” served as reference groups for comparisons. We used a *p*-value < .05 criterion for significance, calculated using the *parameters()* function with the Satterthwaite method.

Data exclusion criteria include: participants with zero variance responses, missing gender or age information, more than two missing questionnaire responses, or aged below 28 or above 55 years. We’ll exclude trials with response times above 4000 ms and participants with over 30% missing trials. Participants aware of the study’s objectives or reporting strategies that negate the prime’s effect were also excluded.

To ensure robustness, we compared models with and without exclusions. The ‘*emmeans*’ [68] package was used to create predicted marginal means, contrasts, and comparisons for fixed effects. All analysis scripts are available in the Online Supplementary Material.

### 2.4 Participants

An initial sample of 278 participants’ responses was collected (see Table 2 for descriptive statistics). We applied the filters specified in the protocol to remove participants and trials. We checked the open response to detect participants which were aware of the study hypothesis, we removed 10 participants (4%). Also, we excluded trials with responses time above 4000, this yield to the exclusion of 7% of trials. Also, we excluded participants which had less than 20 experimental trials after the high response times were removed which removed 32 participants (12%). We did not detect participants’ responses with zero variance. The final sample consisted of 235 participants ranging from 28 to 55 years.

**Table 2:** Descriptive statistics of main variables of interest by condition.

Experimental manipulation	Harshness judgment response	
	Old Condition *M (SD)*	Young Condition *M (SD)*
Condition Age	2.77 (1.81)	2.81 (1.81)
Prime Disgust	2.93 (1.84)	2.74 (1.70)
Prime Sadness	2.91 (1.81)	3.00 (1.88)
Prime Neutral	2.71 (1.77)	2.71 (1.77)
**Scales**	**Participant response to questionnaires**	
	**Old Condition** **M (SD)**	**Young Condition** **M (SD)**
Disgust Sensitivity Score	2.30 (0.37)	2.29 (0.38)
Ageism Scale Score	2.43 (0.60)	2.45 (0.51)
**Sociodemographic information**	**Old Condition** **M (SD)/ %**	**Young Condition** **M (SD)/ %**
Participant’s Age in years	38.40 (8.40)	38.10 (7.27)
Sex: male	52%	31%

Lower scores indicate harsher moral judgment.

Descriptive statistics showed little mean difference between conditions and similar standard deviations (see supplementary online material for statistics for each vignette). Mean responses were about the middle range of response options (see Table 2).

## 3 Results

### 3.1 Preregistered inferential results

**Fig 1:**
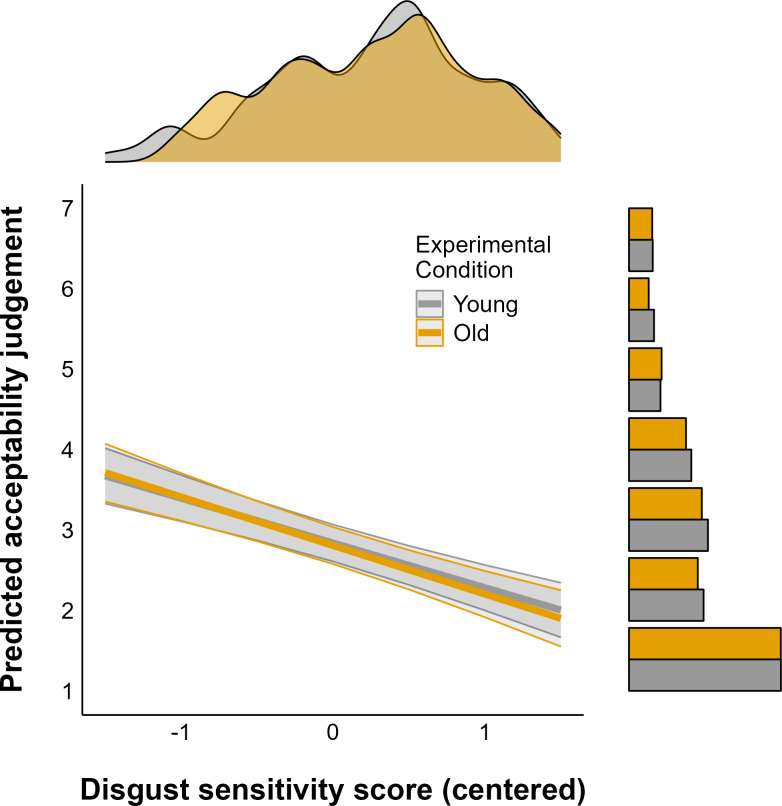
Predicted acceptability judgement as a function of disgust sensitivity score (centered). The relationship is shown for both young (gray) and old (yellow) experimental conditions. Shaded areas are 95% confidence intervals. Marginal plots show the distributions of the predictor (top) and outcome (right) variables for each group.

We strictly followed our preregistered report protocol [69] for the inferential analysis. For inferential purposes we modeled the response (harshness moral judgment) with a linear mixed model effects regression. In the model we included the condition interaction (Young vs Old) with the disgust sensitivity score and condition interaction with prime type. As random effects we included participant and vignette identifiers which produced a convergent model. Regarding the inferential model, we found that the convergent model included item and participant as random effect (see Table 3). Judging by the intraclass correlation coefficient value the mixed model seems to be an adequate model for the data presented. The coefficients indicate a small negative effect of the Old Condition and no significant interaction effect between disgust sensitivity and condition. Specifically, disgust sensitivity led to harsher moral judgments in both conditions (see Fig 1, RQ1). Priming conditions resulted in no effect on the harness of moral judgements (RQ2). All the analysis showed the same pattern when the excluded participants and trials were included as sensitivity check (see online supplementary material for details).

**Table 3:** Coefficients of main model.

Coefficient	*B* 95% CI *β*	*p* value
Fixed effects		
Condition Age [Old]	−0.07 [−0.27, 0.14] −0.03	0.881
Disgust Sensitivity Score	−0.56 [−0.89, −0.22] −0.04	0.001
Condition Age x Disgust Sensitivity Score	−0.05 [−0.53, 0.44] 0.01	0.846
Prime [Sadness]	0.27 [−0.79, 1.33] 0.15	0.617
Prime [Disgust]	0.00 [−1.07, 1.33] 0.00	0.617
Condition Age x Prime [Sadness]	−0.10 [−0.28, 0.07] −0.06	0.244
Condition Age x Prime [Disgust]	0.21 [0.03, 0.38] 0.12	0.019
**Random Effects variance components**	***σ* 95% CI**	
Participant	0.35	
Item	0.60	
Residual	0.74	
Model adjustment indices		
ICC	0.46	
R^2^ Conditional (Fixed and random effects)	0.47	
R^2^ Marginal (Fixed effects)	0.02	

### 3.2 Planned exploratory inferential results

We explored the effects of affective pictures on the harshness of moral judgments, comparing outcomes between conditions featuring old versus young actors. Priming conditions resulted in a positive interaction of disgust prime, this is, disgust prime led to less severe judgements when paired to old themed vignette condition (Table 3).

**Fig 2:**
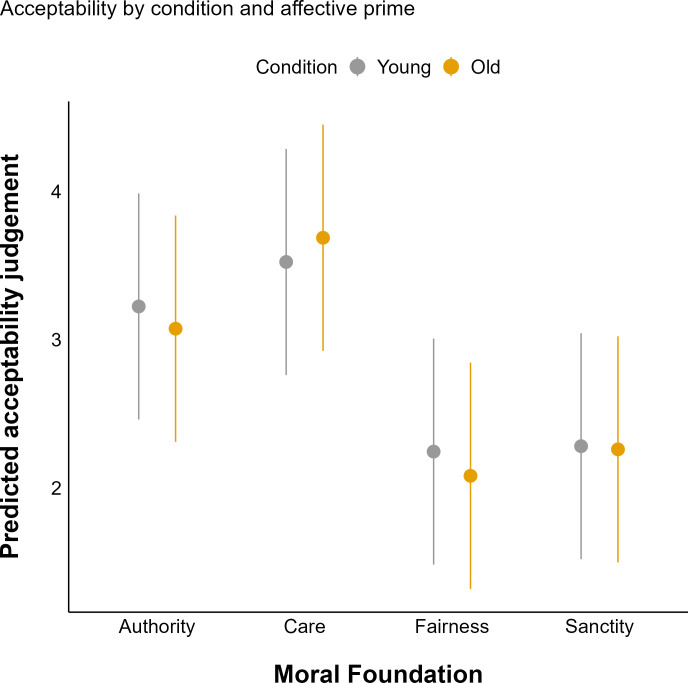
Predicted acceptability judgements for young (gray) and old (yellow) participants across four Moral Foundations. Points are predicted means, and error bars are 95% confidence intervals.

When comparing between conditions (old vs. young), disgust priming facilitates less severe judgments in the old condition (RQ2). This result suggests that the influence of disgust priming on moral judgments is sensitive to the age of the target (see Fig 2). The analysis of interactions and paired contrasts between age groups (young and old) and types of prime stimuli (neutral, sad, and disgust) indicates that differences in reactions to these stimuli are moderated by the participant’s age. No statistically significant difference was found in reactions to neutral and sad stimuli between the young and old conditions (b = −0.10, SE = 0.09, z =−1.165, p = .244). In contrast, the reaction to disgust stimuli compared to neutral was significantly different (b = 0.20, SE = 0.09, z = 2.34, p = .019), showing that the young condition group responds differently to these stimuli compared to the old group. Most notably, the difference between reactions to sad and disgust stimuli was statistically significant (*b* = 0.312, SE = 0.089, *z* = 3.508, *p* = .0005), suggesting greater differential reactivity in the young group to these types of emotional stimuli.

Also, we examined relationships between demographic traits (age, gender) and individual differences (ageism scores, disgust elicited by the word “old”, etc.) with primary outcome measures of judgments. Results showed no effects (see Table 4). Regarding the relationship between ageism scores and emotions elicited by old people, focusing on how this interacts with age condition and affects moral judgment harshness, we did not find any relevant relationship (see Table 4).

**Table 4:** Model comparison for exploratory analysis.

Model	AIC	R² (Cond.)	R² (Marg.)	Model comparison
Baseline model	19054.1	.47	.02	–
Model with individual differences	19060	.48	.02	*χ*²(8) = 11.15, *p* = 0.19
Model with demographics	19058.9	.48	.02	*χ*²(4) = 3.29, *p* = 0.510

*Note:* AIC = Akaike Information Criterion

## 4 Discussion

According to our first research question, which investigates the role of disgust sensitivity in judging two age scenarios, our findings show that individuals with higher disgust sensitivity tend to make more severe moral judgments. This aligns with previous research suggesting that the experience of disgust can influence moral judgments [3,32,70]. The consistent relationship between disgust sensitivity and harshness of moral judgment across conditions underscores the robustness of this effect. However, contrary to our expectations and to previous studies [6], we did not find an interaction effect between disgust sensitivity and the experimental condition. According to these results, the vignette actor’s age has no influence on the effect of disgust sensitivity on moral judgment. Specifically, we found that the impact of disgust sensitivity on moral judgments remains consistent across different experimental contexts (i.e., age of the actor), suggesting that the influence of disgust on moral judgments is more general than initially hypothesized. It is important to note that our study differs from previous investigations in several methodological aspects (see [6]). Firstly, we employed a larger number of vignettes to assess moral judgments, potentially providing a more comprehensive evaluation of moral judgment. Additionally, our sample had an upper age restriction compared to previous studies. Disgust propensity has been shown to decline with age [70–72] so future studies should consider the age of the participants when we examine this effect.

These methodological differences could account for some discrepancies in the results and provide new insights into the disgust-morality relationship. These findings reinforce the notion that emotions, specifically disgust, play a significant role in our moral judgments. This has implications for our understanding of moral decision-making processes and could have applications in fields such as law, ethics, and public policy. The consistent effect of disgust sensitivity across conditions suggests that individual differences in disgust sensitivity might be a reliable predictor of the harshness of moral judgment [30,70].

The strength of the relationship between disgust sensitivity and moral judgments stands in contrast to recent debates regarding the impact of incidental disgust on moral judgments [28–31 but see [25–27]. As Donner and colleagues [70] note, research on disgust sensitivity does not take a stance on whether the causal influence of disgust on moral judgments is driven by disgust that is incidental or inherent to the moral judgment process. Therefore, it may be that people high in disgust sensitivity are more susceptible to experiencing incidental disgust or to misattributing it (or both). Conversely, disgust sensitivity may be indicative of a stronger propensity to experience intrinsic disgust, which may have attitudinal and behavioral moral consequences. Disgust may influence various aspects of social interactions, serving a pivotal function within the context of a preventive “behavioral immune system” [11], which relies on heuristic signals to avert contact with individuals perceived as potentially infectious. For example, individuals who are perceived as disgusting are often judged as more immoral [33].

A second aim of this research was to investigate the effect of affective pictures on the harshness of moral judgments (RQ2). Postulating that disgust is involved in certain aspects of moral judgments [1,3,58], such as how harshly moral violations of old adults are judged [6], we examined whether the influence of affective priming of disgusting pictures on moral judgments was sensitive to the target’s age. When exposed to disgust priming, the comparison between conditions shows that participants’ moral judgments were less harsh in the old condition than in the young condition. Therefore, our results show that the effects of disgust priming on moral judgments were sensitive to the experimental condition, that is, to the target’s age. This finding suggests that the age of the target may constitute a differential factor when making moral judgments when experiencing incidental disgust.

Our findings align with the work of Giner-Sorolla et al. [3] and Uhlmann et al. [34], which suggests that incidental disgust may exert a more significant influence on evaluations of individuals, particularly when morally irrelevant factors are considered. One such factor could be the target’s age. Indeed, Laurent and Li [33] propose that the influence of disgust within the moral domain is more likely to affect perceptions of moral character than judgments of specific moral behaviors. Further, our results are consistent with previous research showing that the direction of affective priming effects on moral judgements depends on certain moderating variables [73]. Altogether, our findings suggest that a fine-grained approach to the effects of incidental emotional stimuli on moral judgments should take account the interaction between the affective quality of the stimuli and specific features of the target (e.g., the age of the target performing the action).

As stated in RQ2, we expected that disgust pictures will have a different effect on the harshness of moral judgments than other affective pictures (sadness). Our results revealed that, unlike disgust priming, the effect of sadness pictures in moral judgments were insensitive to the target’s age. No effect of neutral priming was observed in any condition.

Prior research on the specific role of disgust in morality has shown that, relative to sadness, disgust more strongly influences moral judgments [56]. Other studies found no evidence that incidental sadness influenced moral judgments [74] Disparity between results may be explained by methodological differences, such as the intensity of the affective induction [64]. Our results add to the existing literature by showing that how the target is perceived may determine whether disgust and sadness affective inductions have differential effects on moral judgments.

Interestingly, disgust, induced through affective priming, did not cause harsher moral judgments. This finding is consistent with previous research on affective priming and moral judgments [58,59], suggesting that affective priming may be a particular case of an affective induction paradigm when it comes to moral research. One possibility is that emotional pictures presented at extremely short exposure times may fail to capture the more “cognitive” dimensions of the emotional response. For instance, research on the impact of short exposure times in affective priming indicates that rapid exposure can trigger initial emotional responses, while responses requiring more cognitive effort typically require longer exposure times for elicitation [75]. Since we did not measure the experience of disgust in the participants, this question requires empirical evidence. Future studies should examine whether the effects of different affective pictures on moral judgments are consistent when presented at longer exposure times.

Some limitations should be noted. Firstly, caution is warranted in drawing definitive conclusions about the affective priming effects on moral judgments. As noted earlier, the influence of induced disgust on moral judgments remains a contentious issue, with several studies highlighting the need for further investigation [28–30]. Secondly, we did not include non-moral situations. Therefore, we cannot claim that our results are restricted to moral judgments. While consistent evidence indicates that higher disgust sensitivity is associated with stronger condemnation of moral violations [32,70], this relationship may, in part, reflect a broader connection between disgust sensitivity (or potentially emotional reactivity more generally; [76]) and negative evaluations in social judgments. Our results suggest that the relationship between disgust and moral judgment may remain particularly relevant when considering specific factors, such as the target’s age. In addition, prior research showed that suboptimal affective priming by disgust and horror pictures only influenced moral judgments, with no effect on non-moral dilemmas [58]. This finding was explained as moral judgments recruiting affective information to a greater extent than non-moral judgments. In this vein, we did not measure the affective dimensions (i.e., valence and arousal) of moral dilemmas. It may be the case that the age of the target increases or decreases valence and/or arousal ratings of moral dilemmas, which may be informative in the context of this research. Finally, in this study we measured moral judgments using only self-reported measures. In future studies, it will be recommended to use some implicit measures to check if we find similar results.

In conclusion, this study makes a meaningful addition to the existing literature on disgust and moral judgment by showing the different effects of dispositional and induced disgust. Furthermore, the study highlights the nuanced role of affective priming in moral judgments. It shows that the effects of disgust on moral judgments is sensitive to the age of the target, leading to more lenient judgments when the target is an older individual. This finding suggests that the emotional responses elicited by the prime interact with the perceived characteristics of the moral agent, challenging the notion that disgust universally strengthens moral judgments. In addition, the research emphasizes the importance of considering individual differences in disgust sensitivity when studying moral judgments. The consistent effect of disgust sensitivity across age conditions suggests a broader, more pervasive influence of this trait on moral judgments, reinforcing its potential as a predictor of moral judgment severity.
